# Highly Sensitive and Wide-Range Detection of Thiabendazole via Surface-Enhanced Raman Scattering Using Bimetallic Nanoparticle-Functionalized Nanopillars

**DOI:** 10.3390/bios14030133

**Published:** 2024-03-04

**Authors:** Hyunjun Park, Gayoung Kim, Woochang Kim, Eugene Park, Joohyung Park, Jinsung Park

**Affiliations:** Department of Biomechatronic Engineering, College of Biotechnology and Bioengineering, Sungkyunkwan University, Suwon 16419, Republic of Korea; guswns1105@g.skku.edu (H.P.); kgy9173@g.skku.edu (G.K.); wkim91@skku.edu (W.K.); delorean12@g.skku.edu (E.P.)

**Keywords:** thiabendazole, wide-range detection, bimetallic nanoparticles, nanopillar structure, surface-enhanced Raman scattering

## Abstract

Thiabendazole (TBZ) is a benzimidazole; owing to its potent antimicrobial properties, TBZ is extensively employed in agriculture as a fungicide and pesticide. However, TBZ poses environmental risks, and excessive exposure to TBZ through various leakage pathways can cause adverse effects in humans. Therefore, a method must be developed for early and sensitive detection of TBZ over a range of concentrations, considering both human and environmental perspectives. In this study, we used silver nanopillar structures (SNPis) and Au@Ag bimetallic nanoparticles (BNPs) to fabricate a BNP@SNPi substrate. This substrate exhibited a broad reaction surface with significantly enhanced surface-enhanced Raman scattering hotspots, demonstrating excellent Raman performance, along with high reproducibility, sensitivity, and selectivity for TBZ detection. Ultimately, the BNP@SNPi substrate successfully detected TBZ across a wide concentration range in samples of tap water, drinking water, juice, and human serum, with respective limits of detection of 146.5, 245.5, 195.6, and 219.4 pM. This study highlights BNP@SNPi as a promising sensor platform for TBZ detection in diverse environments and contributes to environmental monitoring and bioanalytical studies.

## 1. Introduction

Thiabendazole (TBZ) is a benzimidazole and is widely used as a fungicide and pesticide [1]. Owing to its potent anti-bacterial and anti-fungal properties, TBZ effectively inhibits the growth of fungi and molds that may occur during storage and distribution of crops [2,3,4]. It is primarily employed to prevent diseases, such as mold and decay in fruits and vegetables and for the surface treatment of banana and orange peels [5,6]. However, extensive application of TBZ can potentially affect human health [7]. Although TBZ effectively mitigates agricultural threats, its chemical stability and solubility in the environment remain concerning. Once TBZ leaches into water resources and beverages, exposure to high concentrations may cause severe health issues, such as liver damage, thyroid hormone imbalance, anemia, and even cancer in humans [8,9,10,11,12]. Due to these risks, the World Health Organization (WHO) has regulated the Acceptable Daily Intake (ADI) of TBZ to be below 0.1 mg/kg (500 nM). Thus, methods for early and sensitive detection of TBZ must be developed.

Conventionally, TBZ is detected using gas chromatography–mass spectrometry (GC–MS) [13], liquid chromatography–mass spectrometry (LC–MS) [14], and high-performance liquid chromatography (HPLC) [15]. However, these techniques are challenging and require expertise, extensive laboratory facilities, and time-consuming pre-processing methods. Attempts to utilize electrochemistry to detect TBZ were unsuccessful because the stable ring structure of TBZ limits its electrochemical activity [16]. Sensors based on surface-enhanced Raman scattering (SERS) have been considered for TBZ detection. The unique vibrations of molecules in ring structures are highlighted in the Raman signals, making SERS particularly suitable for detecting TBZ [17,18,19]. However, SERS-based TBZ detection still encounters limitations while detecting TBZ at extremely low concentrations in environmental and biological samples. Therefore, nanostructured substrates must be developed that are more effectively tailored to achieve a high detection sensitivity.

In the present study, we developed a high-performance SERS substrate by functionalizing nanopillars with bimetallic nanoparticles to achieve a highly sensitive and wide-range detection of TBZ in various environmental and biological samples. We fabricated silver nanopillars (SNPis) via an electrochemical method and Au@Ag bimetallic nanoparticles (BNPs) using a reduction method. Subsequently, BNP was integrated onto SNPi, resulting in the BNP@SNPi substrate displaying highly sensitive detection capabilities through an increased surface area and the generation of multiple hotspots. Characterization using SEM and TEM confirmed the unique composition of the BNP@SNPi substrates. Ultimately, BNP@SNPi demonstrated highly sensitive and wide-range detection of TBZ, successfully identifying TBZ in environmental samples, such as tap water, drinking water, juice, and human serum. These breakthroughs demonstrate the potential of the developed BNP@SNPi substrate as a sensor platform for detecting various targets in diverse environments, contributing substantially to the field of environmental monitoring and bioanalytical studies.

## 2. Material and Methods

### 2.1. Chemical Agents

All chemical reagents (sulfuric acid [98% *w*/*v*], hydrogen peroxide [30% *w*/*v*], potassium dicyanoargentate [KAg(CN)_2_], chloroauric acid [HAuCl_4_], sodium citrate, L-ascorbic acid [AA], silver nitrate [AgNO_3_], ethyl alcohol (99.9%), 4-aminothiophenol [4-ATP], pesticides [thiabendazole (TBZ), malathion, chlorpyrifos, thiacloprid, thiamethoxam], and human serum) were purchased from Sigma Aldrich (St. Louis, MO, USA). Glass slides were purchased from Paul Marienfeld GmbH Co. KG (Lauda-Königshofen, Germany). Tap water was used in the laboratory and drinking water and juice were obtained from a local market. Aqueous solutions were prepared using Millipore deionized (DI) water with a resistivity of 18.2 MΩ cm at 25 °C. Solutions of 4-ATP and pesticide were dissolved in ethyl alcohol.

### 2.2. Synthesis of SNPi, Gold Nanoparticles, and Bimetallic Au@Ag Nanoparticles

We fabricated SNPi according to the method used in our previous studies [20]. Briefly, a thin gold plate was deposited on a glass slide that was pretreated with a piranha solution (H_2_SO_4_/H_2_O_2_ = 3:1) using electron beam evaporation. The electrochemical deposition was performed using a conventional three-electrode system. A 20 mM KAg(CN)_2_ solution was used. SNPi was fabricated at −2.0 V until the electric charge Q reached 120 mC.

Gold nanoparticles (GNPs) were synthesized using the Turkevich method [21]. A solution containing 0.5 mL of 1% (*w*/*w*) HAuCl_4_ and a solution containing 2.5 mL of 1% (*w*/*w*) sodium citrate were added to 50 mL of DI water under stirring and heating. After 30 min, the color of the solution changed to red.

Bimetallic Au@Ag nanoparticles (BNPs) were synthesized following a procedure similar to that reported previously [22,23]. For synthesizing BNP, 2 mL each of GNP, 20 mM AA solution, and 1% sodium citrate solution were added to 40 mL of DI water and stirred gently for 5 min. Then, 600 μL of 10 mM AgNO_3_ was added to the solution with gently stirring. After 15 min, the color of the solution changed to orange.

### 2.3. Optimization of BNP@SNPi SERS Substrate

To optimize the amount of AgNO_3_ during BNP synthesis, BNP was synthesized with various amounts of AgNO_3_ (100–800 μL), following which a 10 μM 4-ATP solution was mixed with each BNP solution at a 1:1 volume ratio. After 20 μL of the mixture was added to each SNPi and dried at room temperature (RT, 23 °C), SERS spectra were confirmed.

To optimize the reaction ratio between the target and BNP solution, the SERS spectrum was checked after mixing 4-ATP and BNP at ratios of 1:1 to 1:5. At this point, the volume of the final mixture was fixed at 2 mL and 4-ATP solutions of various concentrations were prepared so that the 4-ATP concentration of each mixture was also fixed at 10 μM.

### 2.4. SERS Analysis

The SERS spectra were obtained using a Raman microscope (In Via Reflex, Renishaw, Wotton-under-Edge, UK). The wavelength of the laser was 785 nm, and it was focused using a 100× objective lense (Leica DM2700 M, Wetzlar, Germany) and detector (Renishaw Centrus Detector, Wotton-under-Edge, UK). Except for the serum samples, all SERS spectra were measured during 1 s of exposure and three accumulations. Serum samples were measured under the same conditions after 1 s of exposure and after 10 accumulations.

To select the optimal SERS substrate, the SERS spectra of SNPi, SNPi@GNP, and SNPi@BNP were obtained using 4-ATP. Briefly, 10 μM 4-ATP solution was mixed with DI water, GNP solution, and BNP solution at a 1:1 volume ratio, respectively. Then, 20 μL of the mixture was added to each SNPi and dried at 23 °C.

To confirm the performance of the optimized SERS substrate, 4-ATP solutions of various concentrations (1 mM–1 pM) were prepared and the SERS spectra were recorded. After mixing each 4-ATP solution and BNP solution in a ratio of 1:2 according to the optimized method, 20 μL of the mixture was added to SNPi and dried at 23 °C.

TBZ solution (1 mM) was prepared in ethanol and diluted to concentrations ranging from 100 μM to 100 pM. The TBZ solutions (1 mM–100 pM) were mixed with the BNP solution in a 1:2 volume ratio. Subsequently, 20 μL of the mixtures were added onto each SNPi and dried at 23 °C. For the reproducibility test, six different BNP@SNPi SERS substrates were prepared using the same method with 1 mM TBZ. In the selectivity test, 100 μM solutions of pesticides (chlorpyrifos, thiacloprid, thiamethoxam, and malathion) were prepared along with 10 μM of TBZ.

### 2.5. TBZ Detection in Real Samples

Tap water and drinking water samples were filtered once through a syringe filter, and the juice was filtered once through filter paper. A stock solution of 1 mM TBZ was first prepared in ethanol and diluted with filtered tap water, drinking water, juice, and 10% diluted serum for concentration experiments (100 μM–1 nM).

## 3. Results and Discussion

### 3.1. Detection of TBZ in Various Environments Using BNP@SNPi

**Scheme 1:**
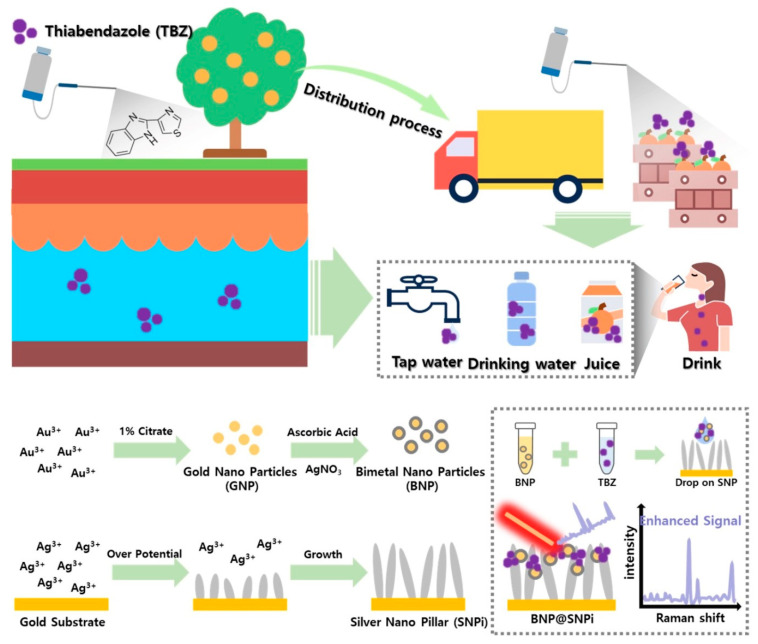
Schematic illustrating various leakage pathways of TBZ and the fabrication and detection strategy for TBZ using the BNP@SNPi SERS substrate.

Scheme 1 illustrates the environments in which TBZ may be present and the strategy for its detection. TBZ applied to the soil as a pesticide or insecticide can flow into groundwater and be present in tap water. Furthermore, it can be applied to fruits and vegetables during distribution. Humans can consume TBZ through drinking water and beverages; consequently, TBZ can be present in the human serum [24]. Therefore, sensitive detection of TBZ in these environments is crucial. We designed a BNP-functionalized SNPi SERS substrate via two strategies. The first strategy uses Au and Ag BNPs. We expected that BNP, which contains both gold and silver components, would ensure stronger Raman signal amplification and chemical stability. In particular, the bimetal particles with gold as the core and silver as the shell are intricately designed to possess abundant electromagnetic hotspots where the target substance can readily adsorb and be immobilized. This unique design induces favorable plasmonic enhancement in SERS signals, resulting in a relatively high Raman intensity for these particles [25]. The second strategy was to use SNPi to produce a substrate that increased the SERS detection sensitivity of TBZ by interacting with BNP. We expected that the interaction of SNPi with BNP would generate extensive hotspots in the three-dimensional space [26,27]. During the preparation, Ag nanopillar-shaped structures were created using electrochemical methods. This involved reducing the silver on the surface of the gold plate by overvoltage using a KAg(CN)_2_ solution [28]. Subsequently, BNP was synthesized, mixed with the prepared TBZ solution in a 1:1 volume ratio, reacted, and applied by dropping it onto the prepared SNPi. Consequently, BNP and SNPi combined with SERS substrates were fabricated and prepared to generate strong and robust SERS signals. The developed BNP@SNPi was then utilized for the highly sensitive and wide-range detection of TBZ in various real environments, such as tap water, drinking water, juice, and human serum.

### 3.2. Characterization of BNP@SNPi Substrate

**Figure 1:**
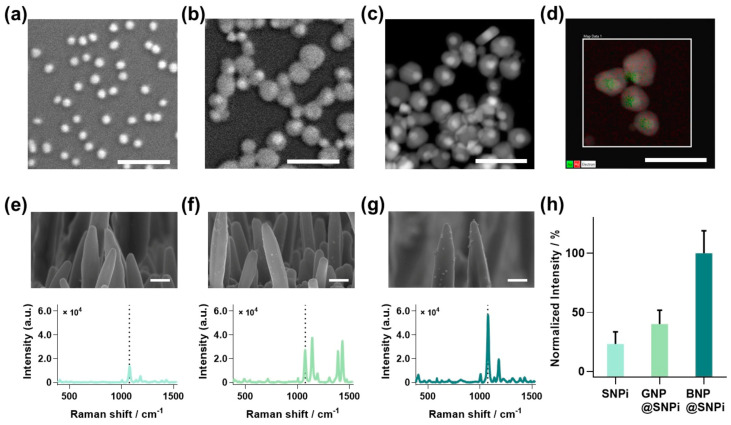
SEM images of synthesized (**a**) GNP and (**b**) BNP, (**c**) TEM images, and (**d**) TEM EDS mapping images of BNP. SEM images based on substrate combinations and Raman intensity of 4-ATP (10 μM) for substrates (**e**) SNPi, (**f**) GNP@SNPi, and (**g**) BNP@SNPi. Dashed lines in the spectra represent the specific Raman peak of 4-ATP at 1076 cm^−1^. (**h**) Normalized Raman intensity data at 1076 cm^−1^ for each substrate.

To fabricate BNP@SNPi, BNP and SNPi were individually prepared using the methods outlined in the experimental section (Figure 1). First, BNPs were synthesized by growing a silver shell on AuNP seeds. The SEM image displays the successful synthesis of GNP seeds with a uniform size (Figure 1a). Subsequently, the silver-shell-coated BNP was characterized for size and core-shell structure using SEM and TEM (Figure 1b,c). Furthermore, the elemental analysis mapping image highlights the distinct composition of the Au core within the green region and the Ag shell within the red region. This offered a visual representation of the amalgamation of these two regions (Figure 1d). Additionally, SNPi was prepared using the optimized conditions from our previous study [29], tailored for SERS-based detection (Figure S1a). Finally, BNP and SNPi were combined to prepare the designed BNP@SNPi structure.

To assess the improved capabilities of the designed BNP@SNPi structure, we compared the SERS performances of bare SNPi, GNP-functionalized SNPi (GNP@SNPi), and BNP-functionalized SNPi (BNP@SNPi). The Raman intensity was measured by reacting each substrate with the representative Raman indicator, 4-ATP, at a concentration of 10 μM. The obtained spectra aligned with the typical Raman spectrum of 4-ATP, clearly revealing peak values of the specific Raman peak at 1076 cm^−1^ (Figure 1e–g). Compared with SNPi, both GNP@SNPi and BNP@SNPi exhibited strong SERS intensities. This enhancement can be attributed to the increased surface area of the substrate owing to the spaces between the nanoparticles and SNPi, facilitating the formation of multiple hotspots [30,31]. Moreover, the enhanced SERS amplification effect of BNP@SNPi, which is superior to that of GNP@SNPi, can be explained by the bimetallic effect, which provides a better electromagnetic field amplification performance than a pure metal [27]. The Raman intensity at the most prominent peak of 1083 cm^−1^, among the specific Raman peaks of 4-ATP, was compared, revealing values of 23.31 ± 10.24, 40.04 ± 11.77, and 100 ± 19 for SNPi, GNP@SNPi, and BNP@SNPi, respectively (Figure 1h). These distinct improvements in the signal intensity demonstrate the potential of BNP@SNPi as a highly sensitive SERS substrate.

### 3.3. Optimization and Sensor Performance of BNP@SNPi SERS Substate

Based on the findings illustrated in Figure 1, we concluded that BNP contributed substantially to shaping BNP@SNPi into a highly effective SERS substrate. Thus, prior to conducting extensive TBZ detection experiments, the BNP@SNPi had to be optimized as a SERS substrate by adjusting the particle form and quantity to achieve the best sensor performance. To explore this, we initially varied the amount of AgNO_3_ solution used to synthesize BNP to control the shell thickness of the particles.

**Figure 2:**
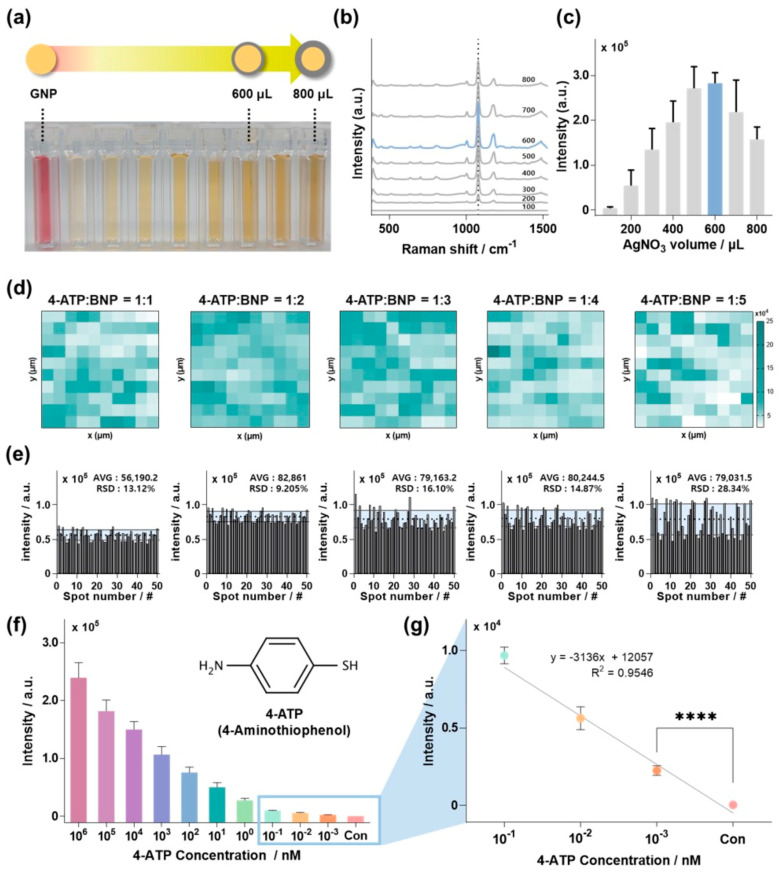
Substrate optimization and performance evaluation. (**a**) Synthesis results of BNP, a crucial component of the BNP@SNPi substrate, based on varying volumes of AgNO_3_ (0 (GNP), 100, 200, 300, 400, 500, 600, 700, and 800 μL, respectively). (**b**) Raman spectra of 10 μM 4-ATP in response to BNP under each condition. (**c**) Raman intensity at 1076 cm^−1^ which is a specific Raman peak of 4-ATP. (**d**) Raman mapping image of the BNP@SNPi SERS substrate according to the reaction ratio of BNP and 4-ATP. (**e**) Raman intensity of 50 random spots in the mapping image. (**f**) Raman intensity at 1076 cm^−1^ according to the concentration of 4-ATP using the optimized BNP@SNPi substrate (inset: molecular structure of 4-ATP). (**g**) Raman intensity and linear equation in the low concentration range of 4-ATP (1 mM–100 pM) (**** *p*-value < 0.0001).

Figure 2a illustrates the gradual thickening of the silver shells of the GNP seeds by adjusting the volume of the AgNO_3_ solution. As the volume of the AgNO_3_ solution increased, the silver shell of the BNP became thicker, the silver content increased, and the color changed to dark yellow. Figure S2 shows the UV–vis absorption spectra of the GNP and BNP synthesized with varying volumes of AgNO_3_ solution. In contrast to GNP, which exhibits a high absorption peak at 520 nm [32], BNP synthesized with accumulated silver shells showed absorption peaks ranging between 410 and 425 nm [33]. Additionally, the presence of the silver shell on BNPs, as confirmed by UV–vis spectroscopy, was further validated using TEM (Figure S3). After preparing the bimetallic nanoparticles with various shell thicknesses, they were mixed with a 10 mM 4-ATP solution and then dropped onto the SNPi substrate for Raman intensity comparison (Figure 2b). Figure 2c shows the Raman intensity at the specific Raman peak of 4-ATP at 1083 cm^−1^ in Figure 2b for each condition. The results indicated that BNP synthesized with 600 μL of AgNO_3_ solution showed the highest performance due to the enhanced SERS through the bimetallic effect, maintaining an appropriate ratio of Au and Ag [34]. In general, as the Ag shell of the BNPs is produced using a AgNO_3_ solution of 600 µL or more, the SERS intensity tends to decrease when surpassing a certain critical threshold [35].

Once the optimal form was determined for BNP by controlling shell thickness, we optimized the quantity of BNP functionalized on SNPi. To achieve this, we manufactured BNP@SNPi SERS substrates by sampling a 1 μM concentration of 4-ATP and the optimized BNP particles in ratios ranging from 1:1 to 1:5 on the SNPi. Subsequently, Raman mapping was performed to analyze the Raman intensity of each BNP@SNPi substrate. Figure 2d shows Raman mapping images corresponding to each reaction ratio. The Raman intensity at the specific Raman peak of 4-ATP (1083 cm^−1^) appears white when low and shifts towards turquoise as it increases at each mapping image. The comparative results revealed that the samples with a reaction ratio of 1:2 between 4-ATP and BNP particles exhibited the highest proportion of turquoise. To quantitatively analyze this, the Raman intensity values from each of the 50 spots are shown in Figure 2e. The mean and standard deviation of the Raman intensity for each condition were 56,190.2 ± 7373.11 (relative standard deviation, RSD: 13.12%), 82,861 ± 7627.05 (RSD: 9.205%), 79,163.2 ± 12,748.2 (RSD: 16.10%), 80,244.5 ± 11,935.6 (RSD: 14.87%), and 79,031.5 ± 22,394.7 (RSD: 28.34%), respectively. In conclusion, the reaction ratio of 1:2 between 4-ATP and BNP particles showed the strongest Raman intensity and uniform standard deviation. As the concentration and quantity of nanoparticles per unit area increased, spontaneous particle aggregation occurred, surpassing the specific threshold of the SERS hotspots [36]. This led to an overall decrease in the SERS intensity and an increase in the standard deviation.

Finally, before detecting TBZ, we validated the accuracy, reliability, and calculating enhancement factor (EF) of the BNP@SNPi substrate using 4-ATP. The detection range of 4-ATP was from 1 mM to 1 pM. Figure 2f depicts the Raman intensity of 4-ATP at the specific Raman peak of 1083 cm^−1^ at various concentrations. The Raman intensity decreased proportionally as the concentration of 4-ATP decreased. Figure 2g shows the Raman intensity at low concentrations of 100, 10, and 1 pM compared to the control scaled accordingly. The linear equation in the provided graph was y = −3136x + 12,057 (R^2^ = 0.9657), and the limit of detection (LOD) of 4-ATP was 103 fM, calculated using the three-sigma formula, LOD = 3.3 × standard deviation/slope. Based on these findings, we computed the analytical enhancement factor (AEF) for the AF–SERS substrate using the following formula [37]:

(1) $$AEF=\frac{I_{SERS}/C_{SERS}}{I_{OR}/C_{OR}}$$

Here, *I_SERS_* represents the SERS intensity, *I_OR_* denotes the SERS intensity observed on the bare substrate, *C_SERS_* stands for the concentration of the Raman indicator on the SERS substrate (i.e., LOD), and *C_OR_* is the concentration of the Raman indicator on the bare substrate. Raman spectra for each condition required for calculation can be found in Figure S4. As a result, the enhancement factor (EF) value for the BNP@SNPi substrate developed by our research team was 6.34 × 10^9^. These findings unequivocally validate the exceptional sensitivity of the proposed sensor in detecting TBZ.

### 3.4. Detection of TBZ Using BNP@SNPi

**Figure 3:**
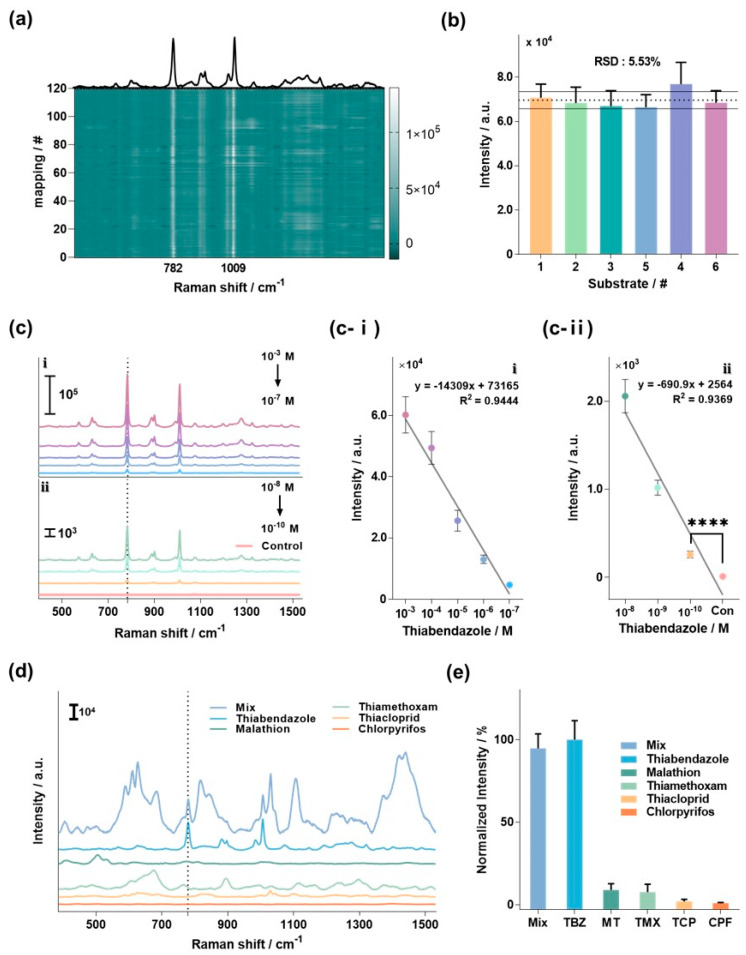
Assessment of specific TBZ detection performance using the BNP@SNPi substrate. (**a**) Uniformity evaluation of TBZ 1 mM on a single fabricated substrate. Highlighted by white lines are areas of high Raman intensity alongside the Raman spectrum of TBZ. (**b**) Reproducibility evaluation of TBZ 1 mM across six distinct fabricated substrates (RSD: 5.53%). (**c**) TBZ detection results based on concentration Raman spectra; Raman intensity and linear equation in the (**c**-**i**) high and (**c-ii**) low concentration ranges (**** *p*-value < 0.0001). (**d**) Raman spectra of various pesticide residues, including TBZ (10 µM for TBZ and 100 µM for the other substances). (**e**) Graph analyzing the selectivity results by normalizing the Raman intensity at 782 cm^−1^, the specific Raman peak of TBZ.

To validate the suitability of the optimized BNP@SNPi for TBZ detection, a series of tests, including reproducibility, sensitivity, and selectivity, were conducted (Figure 3). Figure 3a shows the average Raman spectrum measured by selecting 120 random spots on a single BNP@SNPi substrate reacted with TBZ, and the Raman intensity of each spectrum is presented as a heatmap. The bright line at 782 and 1009 cm^−1^ represents the specific Raman peak of TBZ. The exceptional uniformity across these 50 spots underscores the outstanding performance of the substrate in providing a consistent and reproducible signal, which is essential for reliable TBZ detection. In addition, we evaluated the reproducibility of the six BNP@SNPi substrates exposed to 1 mM TBZ (Figure 3b). Comparing the SERS intensity at the specific Raman peak of TBZ (782 cm^−1^) among these substrates revealed outstanding reproducibility, as indicated by an RSD value of 5.53%.

Next, we assessed the sensitivity of BNP@SNPi for TBZ detection by reacting it with various concentrations of TBZ (1 mM–100 pM), and thoroughly evaluating the detection performance across a range of concentrations. The experimental results are shown in Figure 3c, which illustrates the Raman spectra corresponding to the TBZ concentration in BNP@SNPi. A change in the Raman intensity proportional to the concentration was clearly observed at the specific Raman peak of TBZ at 782 cm^−1^. Region i represents the Raman spectra in the high concentration range (1 mM–100 nM), while region ii represents the spectra in the low concentration range (10 nM–100 pM). In region i, the linear equation for TBZ concentration was calculated as I*_HC_* = −14,744X + 74,846 (R^2^ = 0.972; where X is the TBZ concentration (M)) (Figure 3c-i). Moreover, in region ii, the linear equation was determined as I*_LC_* = −690.9X + 2564 (R^2^ = 0.9369) (Figure 3c-ii). The LOD for TBZ was determined to be 1.06 pM using the following formula: LOD = 3.3 × standard deviation/slope of the regression curve. These results confirmed the excellent sensitivity of BNP@SNPi, indicating its potential to detect TBZ effectively, even under real environmental conditions.

Finally, we assessed the ability of BNP@SNPi to selectively detect TBZ by analyzing the Raman signals of several commonly used residual pesticides in agriculture. The selected pesticides were TBZ, malathion (MT), thiamethoxam (TMX), thiacloprid (TCP), chlorpyrifos (CPF), and a mixed sample (mix) containing all the aforementioned pesticides. At this point, TBZ was tested with a concentration of 10 μM, the other substances were experimented with at a concentration of 100 μM. Figure 3d presents the Raman spectra results for each sample, highlighting a distinct and robust Raman intensity at the specific TBZ Raman peak of 782 cm^−1^ in TBZ and mix. The other target molecules did not exhibit peaks that overlapped with those of TBZ. As previously mentioned in the Introduction, the molecular vibrations of the benzene ring, a constituent of thiabendazole (TBZ), are highlighted, making Raman spectroscopy an advantageous method for TBZ detection. Furthermore, the thiazole moiety composing TBZ efficiently adsorbs to the silver surface, enhancing the detection sensitivity of TBZ [38,39]. In an additional analysis focused on the intensity at 782 cm^−1^, normalization was performed based on the intensity of the TBZ sample. Setting the Raman intensity in the TBZ sample as 100%, the Mix sample and individual components (MT, TMX, TCP, and CPF) showed values of 94.67 ± 8.83%, 8.81 ± 3.78%, 7.50 ± 4.79%, 1.86 ± 1.32%, and 0.87 ± 0.36%, respectively (Figure 3e). This rigorous comparison underscores the specificity of the proposed BNP@SNPi-based TBZ sensor and demonstrates its ability to selectively detect TBZ even at relatively high concentrations of other widely used residual pesticide components.

### 3.5. TBZ Detection in Environmental and Biological Samples

**Figure 4:**
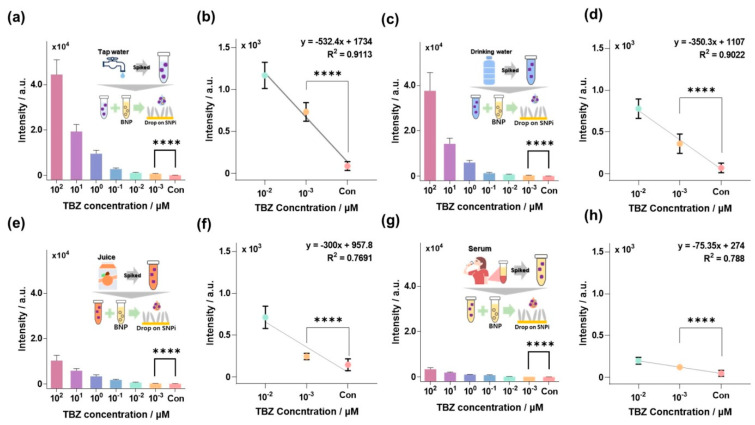
Assessment of TBZ detection in authentic samples with potential TBZ leakage routes. (**a**) Raman intensity at 782 cm^−1^ corresponding to the concentration of TBZ spiked in tap water using the BNP@SNPi substrate. (**b**) Raman intensity and linear equation for low-concentration TBZ (**** *p*-value < 0.0001). (**c**) Raman intensity of TBZ spiked in drinking water under identical conditions. (**d**) Raman intensity and linear equation for low-concentration TBZ (**** *p*-value < 0.0001). (**e**) Raman intensity at 782 cm^−1^ related to the concentration of TBZ spiked in orange juice using the BNP@SNPi substrate. (**f**) Raman intensity and linear equation for low-concentration TBZ (**** *p*-value < 0.0001). (**g**) Raman intensity at 782 cm^−1^ in relation to the concentration of TBZ spiked in human serum using the BNP@SNPi substrate. (**h**) Raman intensity and linear equation for low-concentration TBZ (**** *p*-value < 0.0001).

Experiments were designed to assess the TBZ detection performance of the BNP@SNPi SERS substrate in environmental and biological samples, such as tap water, drinking water, orange juice, and human serum, in which TBZ presence is highly probable (Figure 4). Sample preparation methods are detailed in the Material and Methods section and are briefly illustrated in the inset image in Figure 4.

TBZ is extensively used during cultivation; therefore, it could potentially leak and mix into tap water and drinking water. TBZ was detected using BNP@SNPi-based SERS (Figure S5a,b). Figure S5a,b displayed a distinct SERS peak at approximately 782 cm^−1^, corresponding to the concentration of TBZ. For quantitative analysis, the Raman intensity of the 782 cm^−1^ peak in tap water was represented in Figure 4b. The linear equation calculated in the low-concentration range at the nanomolar level was I_Tap water_ = −873.4X + 3395, and the Limit of Detection (LOD) calculated based on 3-Sigma was 146.5 pM (Figure 4b). Similarly, in Figure 4c, the Raman intensity at 782 cm^−1^, corresponding to the concentration of TBZ in drinking water, is shown. The Raman intensity of the drinking water sample also demonstrated a proportional change with the TBZ concentration. Additionally, the linear equation calculated in the low concentration range in the nanomolar level is I_Drinking water_ = −419.7X + 1684, and the LOD calculated based on 3-Sigma is 245.54 pM (Figure 4d).

Another potential route for TBZ absorption into the human body is via ingesting residual TBZ in fruits, which are present in juices consumed by humans. Furthermore, the ingested TBZ is expected to bind to human serum albumin and could remain in the body [40]. Therefore, the detection performance of the developed SERS substrate had to be evaluated for TBZ in both juices and human serum. The Raman spectra corresponding to the TBZ concentration in orange juice and serum are shown in Figure S5c and S5d, respectively. Subsequent quantitative analysis of Raman intensity at 782 cm^−1^ was conducted, the results of which are shown in Figure 4e and Figure 4g, respectively. Unlike tap water and drinking water, orange juice, and serum, which are unclear samples with numerous impurities, such as pigments and various proteins, exhibited relatively low Raman intensity [41]. Nevertheless, they demonstrated linear concentration changes in the nanomolar range. The linear equations for orange juice and human serum were I_Orange juice_ = −809.9X + 2149 and I_Serum_ = −233X + 874.7, with detection limits of 195.63 and 219.4 pM, respectively (Figure 4f,h). These results indicate the superior performance of BNP@SNPi, characterized by its high sensitivity and wide detection range, surpassing that of previously reported TBZ detection studies (Table S1).

## 4. Conclusions

We developed an advanced BNP@SNPi substrate that integrates SNPi and BNP and is designed to maximize the surface area and SERS hotspots. The performance of the fabricated BNP@SNPi was optimized and evaluated, and it exhibited robust SERS signals in various validations, substantiating its suitability as an SERS substrate. Notably, the BNPs@SNPi substrate used for TBZ detection demonstrated high repeatability, sensitivity, and selectivity. To assess the practical applicability of the sensor, we detected TBZ in various environmental and biological samples. We successfully identified TBZ in tap water, drinking water, orange juice, and human serum with LODs of 146.5, 245.5, 195.6, and 219.4 pM, respectively. Moreover, TBZ detection across a wide concentration range further confirmed the efficacy of BNP@SNPi, offering a comprehensive solution to detecting TBZ in complex and varied environments. This broad applicability underscores the potential of the BNP@SNPi substrate as a versatile tool for environmental monitoring and bioanalytical studies, addressing the critical need for early and sensitive detection of TBZ across diverse samples.

## Data Availability

Data are contained within the article and Supplementary Materials.

## Supplementary Materials

The following supporting information can be downloaded at: https://www.mdpi.com/article/10.3390/bios14030133/s1, Figure S1. SEM image for examining overall morphology of (a) SNPi, (b) GNP@SNPi, and (c) BNP@SNPi (Scale bar: 1μm); Figure S2. UV-vis spectrum of BNP synthesized with GNP and varying volumes of AgNO_3_.; Figure S3. TEM image of BNP for (a) 600 μL and (b) 800 μL of AgNO_3_ (Scale bar: 50 nm); Figure S4. Raman spectrum of (a) 1mM of 4-ATP on bare Au plate and (b) 1 pM of 4-ATP on BNP@SNPi substrate; Figure S5. Raman spectral data for TBZ concentration in (a) tap water, (b) drinking water, (c) orange juice, and (d) human serum; Table S1. Comparison of the SERS sensing performance of the BNP@SNPi substrate and other sensing platforms for TBZ. Refs. [5,42,43,44,45,46,47,48] are cited in Supplementary Materials.
