# Supplementary material for: Highly Sensitive and Wide-Range Detection of Thiabendazole via Surface-Enhanced Raman Scattering Using Bimetallic Nanoparticle-Functionalized Nanopillars
Source: Biosensors (Basel). 2024 Mar 4;14(3):133. doi: 10.3390/bios14030133 (PMC10968244; doi:10.3390/bios14030133)
Supplement: Supplementary file 1 [file biosensors-14-00133-s001.zip › biosensors-2871152-supplementary.pdf]

biosensors

MDPI

Supplementary Materials

Highly Sensitive and Wide-Range Detection of Thiabendazole via Surface-Enhanced Raman Scattering Using Bimetallic Nanoparticle-Functionalized Nanopillars

Hyunjun Park †, Gayoung Kim †, Woochang Kim †, Eugene Park, Joohyung Park * and Jinsung Park *

Department of Biomechatronic Engineering, College of Biotechnology and Bioengineering, Sungkyunkwan University, Suwon 16419, Republic of Korea; guswns1105@g.skku.edu (H.P.); kgy9173@g.skku.edu (G.K.); wkim91@skku.edu (W.K.); delorean12@g.skku.edu (E.P.)

* Correspondence: parkjoodori@skku.edu (J.P.) and nanojspark@skku.edu (J.P.)

† These authors contributed equally to this work.

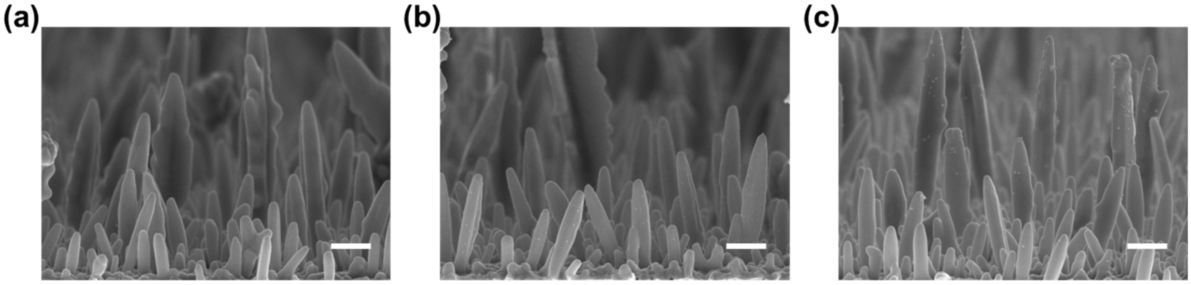

Figure S1. SEM image for examining overall morphology of a) SNPi, b) GNP@SNPi, and c) BNP@SNPi (Scale bar: 1μm).

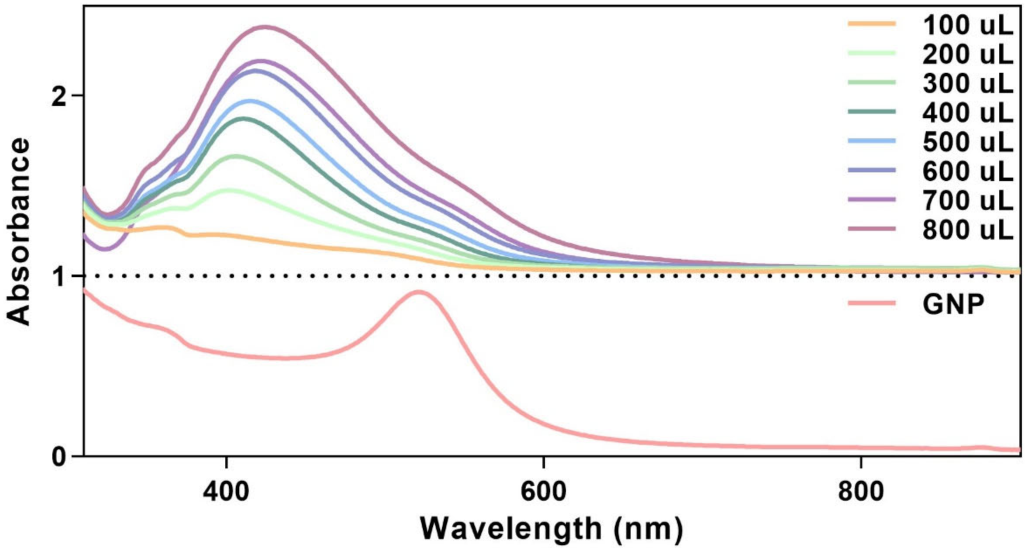

Figure S2. UV-vis spectrum of BNP synthesized with GNP and varying volumes of $AgNO_3$.

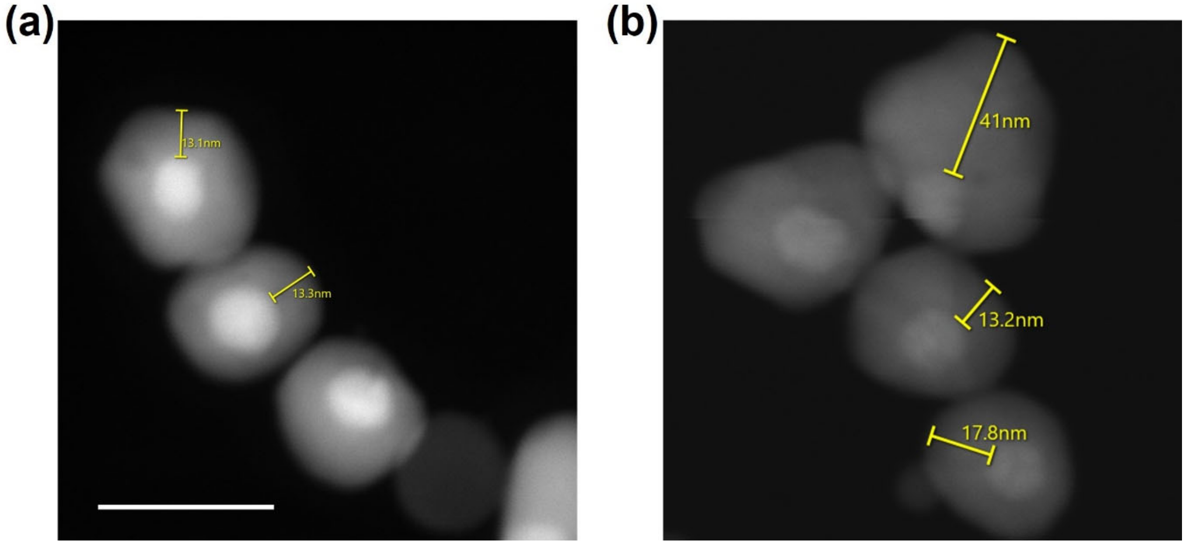

Figure S3. TEM image of BNP for a) 600 μL and b) 800 μL of $AgNO_3$ (Scale bar: 50 nm).

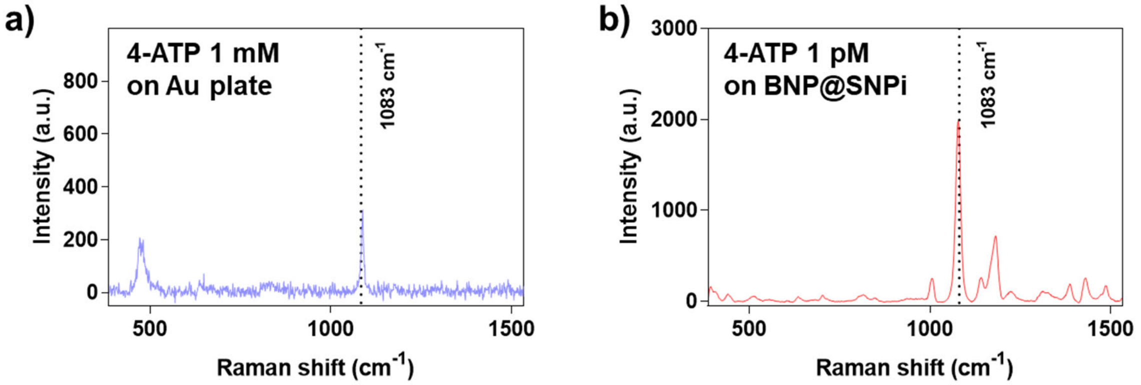

Figure S4. Raman spectrum of a) 1mM of 4-ATP on bare Au plate and b) 1 pM of 4-ATP on BNP@SNPi substrate.

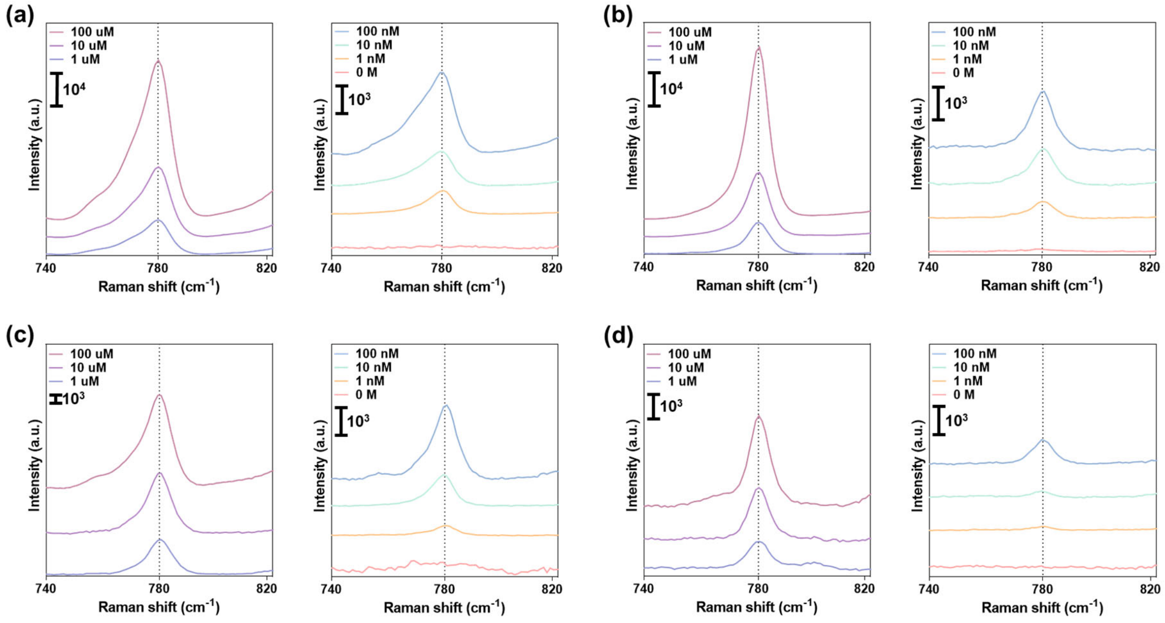

Figure S5. Raman spectral data for TBZ concentration in a) tap water, b) drinking water, c) orange juice, and d) human serum.

Table S1. Comparison of the SERS sensing performance of the BNP@SNPi substrate and other sensing platforms for TBZ.

SERS substrate	Detection range	Detection Limit	References
HAu/Ag nanostar	497 µM – 4.97 nM	4.97 nM	[1]
MOF	9.94 µM – 124 nM	124 nM	[2]
Au/Ag nanopillar	10 µM – 100 pM	100 pM	[3]
AuNR	4.97 µM – 497 nM	497 nM	[4]
HSM@AuNP	497 µM – 24.85 nM	24.85 nM	[5]
AgNF	14.91 – 1.49 mM	1.49 mM	[6]
AgNP paper	497 µM – 497 nM	497 nM	[7]
AgNW-tape	4.97 mM – 49.7 nM	49.7 nM	[8]
BNP@SNPi	1 mM – 100 pM	1.06 pM	**This work**

References

1. Hassan, M.M.; Xu, Y.; He, P.; Zareef, M.; Li, H.; Chen, Q. Simultaneous Determination of Benzimidazole Fungicides in Food Using Signal Optimized Label-Free HAu/Ag NS-SERS Sensor. *Food Chem* **2022**, *397*, 133755, doi:10.1016/J.FOODCHEM.2022.133755.
2. Sun, Y.; Yu, X.; Hu, J.; Zhuang, X.; Wang, J.; Qiu, H.; Ren, H.; Zhang, S.; Zhang, Y.; Hu, Y. Constructing a Highly Sensitivity SERS Sensor Based on a Magnetic Metal-Organic Framework (MOF) to Detect the Trace of Thiabendazole in Fruit Juice. *ACS Sustain Chem Eng* **2022**, *10*, 8400–8410, doi:10.1021/ACSSUSCHEMENG.2C01377/ASSET/IMAGES/LARGE/SC2C01377_0007.JPEG.
3. Ansah, I.B.; Lee, S.H.; Mun, C.W.; Yang, J.Y.; Park, J.; Nam, S.Y.; Lee, S.; Kim, D.H.; Park, S.G. Nanoscale Crack Generation of Au/Ag Nanopillars by in Situ Galvanic Replacement for Sensitive, Label-Free, and Rapid SERS Detection of Toxic Substances. *Sens Actuators B Chem* **2023**, *379*, 133172, doi:10.1016/J.SNB.2022.133172.
4. Pan, H.; Ahmad, W.; Jiao, T.; Zhu, A.; Ouyang, Q.; Chen, Q. Label-Free Au NRs-Based SERS Coupled with Chemometrics for Rapid Quantitative Detection of Thiabendazole Residues in Citrus. *Food Chem* **2022**, *375*, 131681, doi:10.1016/J.FOODCHEM.2021.131681.
5. Qu, Q.; Zeng, C.; Peng, X.; Qi, W.; Wang, M. Sensitive SERS Detection of Pesticide Residues in Beverages Based on an Extraction Integrated Plasmonic Platform. *Sens Actuators B Chem* **2023**, *376*, 133042, doi:10.1016/J.SNB.2022.133042.
6. Li, H.; Luo, X.; Haruna, S.A.; Zhou, W.; Chen, Q. Rapid Detection of Thiabendazole in Food Using SERS Coupled with Flower-like AgNPs and PSL-Based Variable Selection Algorithms. *Journal of Food Composition and Analysis* **2023**, *115*, 105016, doi:10.1016/J.JFCA.2022.105016.
7. He, Z.H.; Zhu, W.W.; Jiang, Y.L.; Zhao, S.S.; Yan, J.; Tan, X.C. Green Synthesis of Paper-Based SERS Substrate for the Quantitative Detection of Thiabendazole by Wipe Sampling. *Microchemical Journal* **2024**, *197*, 109729, doi:10.1016/J.MICROC.2023.109729.
8. Dai, X.; Xue, D.; Liu, X.; Gu, C.; Jiang, T. An Adhesive SERS Substrate Based on a Stretched Silver Nanowire-Tape for the in Situ Multicomponent Analysis of Pesticide Residues. *Analytical Methods* **2023**, *15*, 1261–1273, doi:10.1039/D3AY00022B.
